# Circulating tumour-derived nucleic acids in cancer patients: potential applications as tumour markers

**DOI:** 10.1038/sj.bjc.6603625

**Published:** 2007-02-20

**Authors:** K C A Chan, Y M D Lo

**Affiliations:** 1 Department of Chemical Pathology, The Chinese University of Hong Kong, Prince of Wales Hospital, Shatin, New Territories, Hong Kong Special Administrative Region, China; 2 Li Ka Shing Institute of Health Sciences, The Chinese University of Hong Kong, Prince of Wales Hospital, Shatin, New Territories, Hong Kong Special Administrative Region, China; 3 State Key Laboratory in Oncology in South China, The Chinese University of Hong Kong, Prince of Wales Hospital, Shatin, New Territories, Hong Kong Special Administrative Region, China

**Keywords:** tumour marker, plasma/serum DNA, plasma/serum RNA, circulating nucleic acids

## Abstract

Tumour-associated changes have been observed in the circulating nucleic acids of cancer patients and have been proposed to be useful for the detection and monitoring of cancers. In this review, different approaches for detecting tumour-associated nucleic acids in the circulation and their potential applications as tumour markers are discussed.

Despite the advances in anticancer therapies, cancer remains a leading cause of death in developed countries. For most cancers, the extent of disease at the time of diagnosis is an important factor for determining treatment outcome. In this regard, tumour markers have been developed for the purpose of screening for early malignant diseases. Besides, they are also valuable in monitoring cancer progression as well as prognostication. Existing markers are mainly proteins produced by tumour cells. Unfortunately, only a subset of cancers would secrete specific proteins that can be used as tumour markers. In contrast to tumour-associated proteins, genetic aberrations are frequently observed in cancers and some of these changes are believed to be directly involved in carcinogenesis. With the developments of molecular techniques, such changes can also be detected in the plasma or serum of cancer patients and, thus, may open up the potential of utilising circulating nucleic acids as a new generation of tumour markers. This review summarises the different approaches for detecting tumour-associated nucleic acids in the circulation and their potential applications as tumour markers.

## APPROACHES FOR DETECTING CANCER-ASSOCIATED CHANGES IN CIRCULATING NUCLEIC ACIDS

### Level of total circulating DNA

The existence of extracellular nucleic acids in the circulation was first reported by Mandel and Metais in 1948. However, owing to the technological limitations at that time, further development came only after 30 years when Leon *et al* demonstrated that cancer patients had much higher circulating DNA concentrations than those suffering from non-malignant diseases (Leon *et al*, 1977). Moreover, they showed that, in some cases, the levels of circulating DNA would decrease after successful anticancer therapy (Leon *et al*, 1977). These interesting findings have suggested the potential application of circulating cell-free DNA level as a surrogate marker for cancers. Recently, Gautschi *et al* (2004) demonstrated that plasma DNA level was significantly associated with cancer stage in patients with non-small-cell lung cancers. Patients with more advanced disease had significantly higher levels of circulating DNA (Gautschi *et al*, 2004). In addition, they also showed that plasma DNA levels were of prognostic value for predicting survival in patients undergoing chemotherapy. Patients with higher circulating DNA levels, either at baseline or after treatment, had significantly reduced probability of long-term survival (Gautschi *et al*, 2004). Interestingly, in a prospective study by Gormally *et al* (2004), who followed up more than 500 000 healthy individuals from 22 centres, the elevation of circulating DNA level was shown to be a risk factor for future development of leukaemia. Among these over 500 000 subjects, 359 of them developed cancers after a mean follow-up of 89 months. The plasma DNA levels of those subjects who subsequently developed cancers were compared to the levels of 776 matched subjects who had not developed cancer, and it was demonstrated that subjects with elevated plasma DNA at baseline had increased risk of developing leukaemia in the next 36 months (Gormally *et al*, 2004).

The precise mechanisms leading to the elevated plasma DNA levels in cancer patients are not clear. One hypothesis is the direct release of tumoral DNA into the blood from the rapidly turning-over cancer cells. Diagnostically, the elevation of circulating DNA is not specific for malignant conditions and plasma DNA levels are also increased in other physiological and disease conditions, for example pregnancy and trauma. Thus, methods for the detection of tumour-specific DNA changes have been developed.

### Tumour-associated mutations

Mutations in oncogenes and tumour suppressor genes are commonly detected in cancers and these changes are important in the pathogenesis of cancers. Therefore, such mutations can be recognised as a signature of tumour-derived DNA. In 1994, Sorenson *et al* (1994) detected *KRAS* gene mutations in the plasma and tumours of three patients suffering from pancreatic cancer. Their findings provided direct evidence for the presence of tumour-derived DNA in the circulation of cancer patients. Recently, studies have also reported that tumour-associated mutations could be found in the plasma/serum of individuals exposed to carcinogens, and the presence of such mutations in the circulation may indicate increased risk of developing cancers (Gormally *et al*, 2006; Hagiwara *et al*, 2006). Through the detection of common *p53* mutations at codons 273, 249 and 248, Hagiwara *et al* (2006) reported that *p53* mutations were readily detectable in the plasma of some smokers without cancer but not in the plasma of non-smokers. Furthermore, they demonstrated that the frequency of *p53* mutations were associated with years of smoking. Their results suggested that the presence of *p53* mutations in plasma DNA could reflect the exposure to carcinogens and, hence, the chance of developing lung cancer. Their hypothesis is further supported by a longitudinal prospective study by Gormally *et al* (2006),which detected mutations of the *KRAS2* and the *p53* genes in 1.2 and 3.6%, respectively, of healthy individuals. The presence of such mutations in plasma was shown to be associated with an increased risk of developing bladder cancer (Gormally *et al*, 2006).

Technically, allele-specific polymerase chain reaction (PCR) is commonly applied for the detection of tumour-associated mutations in the plasma/serum of cancer patients. This method allows the detection of a mutated sequence present at a low concentration among a background of wild-type sequences. However, the design of allele-specific primers requires the sequence information of the mutation, which can only be obtained when the primary tumour tissue is available. Alternatively, hotspot-mutations, for example mutations at codons 248 and 249 of the *p53* gene and mutations at the codon 12 of the *KRAS* gene, can be screened for in the plasma (Gormally *et al*, 2006; Hagiwara *et al*, 2006). However, this approach would miss the tumour-associated mutations occurring outside the mutation hot spots and, hence, might reduce the sensitivity of the test. Owing to these technical difficulties, detection of tumour-associated mutations in the plasma/serum has not been extensively used as a clinical tool for cancer detection. The recently introduced mass spectrometry-based method, the single allele base extension reaction (SABER), has been shown to be particularly useful for the detection of single-nucleotide changes in a background of wild-type sequences (Ding *et al*, 2004). For example, it can effectively identify fetal-derived single-nucleotide mutations of the *beta-globin* gene in the plasma of pregnant women (Ding *et al*, 2004). This technique could also be applied for the detection of tumour-associated single-nucleotide mutations in the plasma of cancer patients.

### Microsatellite alterations

Microsatellite alterations, for example, loss of heterozygosity (LOH), are frequently demonstrated in tumour tissues. The detection of LOH in plasma/serum was reported in 1996 in patients suffering from small cell lung cancer (Chen *et al*, 1996). The microsatellite alterations observed in the plasma were identical to those found in the primary tumours of the respective patients, thus indicating their tumoral origin (Chen *et al*, 1996). Their findings were particularly remarkable as they indicated that a major proportion of the circulating DNA in these cancer patients was derived from tumour cells.

Although the presence of microsatellite alterations in plasma/serum are more commonly found in patients with invasive tumours, regional spread and distant metastases, these changes have also been demonstrated in patients with localised and small tumours (Eisenberger *et al*, 2006). These results indicate that the amount of DNA released from the tumour can be substantial even in the early stage of the disease.

Technically, as the detection rate of LOH with a single microsatellite marker is relatively low, a panel of microsatellite markers is usually required for the detection of LOH in tumour tissues and plasma/serum to achieve the necessary sensitivity.

### Viral DNA

Virus infection has been implicated in the pathogenesis of several types of cancers. Thus, it is possible to use circulating viral nucleic acids as a tumour marker for the detection and monitoring of these virus-associated cancers. In this regard, the use of circulating Epstein–Barr virus (EBV) DNA in nasopharyngeal carcinoma (NPC) has been the most extensively studied and has appeared to show much promise.

In 1999, Lo *et al* (1999) reported that, using real-time PCR, EBV DNA could be detected in the plasma of 96% of NPC patients but only in 7% of healthy subjects. In addition, they showed that the levels of circulating EBV DNA could reflect tumour load. Patients with advanced stage disease had a higher plasma/serum EBV DNA than those with early disease (Lo *et al*, 1999). Later, it was further demonstrated that the concentration of plasma EBV DNA correlated with tumour load in an animal model (Chan *et al*, 2005a). In subsequent studies, circulating EBV DNA levels, either at the time of diagnosis or after radiotherapy, have been shown to be an important prognostic factor for patients' survival, independent of cancer stage (Lo *et al*, 2000; Chan *et al*, 2002). In view of its prognostic value, circulating EBV DNA analysis has been proposed to be incorporated in the risk stratification system for NPC to complement the TNM staging system (Leung *et al*, 2006).

Taking advantage of the robustness of plasma EBV DNA analysis, the elimination kinetics of tumoral DNA was studied in NPC patients. It was shown that the median half-life for the clearance of plasma EBV DNA after surgical resection of tumours was only 138 min (To *et al*, 2003). This surprisingly short half-life of circulating EBV DNA implies that large amounts of tumoral DNA are being constantly released into the circulation in NPC patients. It is estimated that over 3 000 000 EBV genomes are being released into the plasma of a NPC patient in each hour to maintain a plasma EBV DNA level of 6000 EBV genome per millilitre, which is the median level for early-stage NPC patients (Lo *et al*, 1999).

These findings raised the question whether active viral replication would be responsible for producing the large amounts of EBV DNA in the plasma of NPC patients. Through ultracentrifugation and DNase treatment of plasma/serum, it was shown that circulating EBV DNA in NPC patients was not associated with intact virions and, hence, did not support the presence of active viral replication (Chan *et al*, 2003). Consistent with these data is the fact that circulating EBV DNA molecules were mainly short DNA fragments of less than 200 bp (Chan *et al*, 2003).

In contrast to the detection of tumour-associated mutations and microsatellite alterations, the detection and quantification of viral DNA in human plasma by real-time PCR is relatively simple, accurate and fast. The whole process from blood collection to PCR analysis can be completed in 3 h. Therefore, plasma viral DNA analysis has a high potential for clinical application. In fact, quantitative EBV DNA analysis has been adopted by several centres as a routine clinical test for the detection, monitoring and prognostication of NPC.

### Hypermethylation of tumour suppressor genes

In addition to tumour-derived genetic changes, tumour-associated epigenetic alterations have also been reported in the plasma or serum of cancer patients. DNA methylation is an epigenetic characteristic associated with the silencing of gene expression. Hypermethylation of tumour suppressor genes is an important mechanism in the pathogenesis of cancer and has been reported in a wide range of cancers. To date, aberrant methylation of tumour suppressor genes has been detected in plasma/serum of patients suffering from many cancers (Hoque *et al*, 2006; Bazan *et al*, 2006). Although these changes are more commonly found in patients with more advanced disease, the simultaneous detection of aberrant methylation of multiple tumour suppressor genes has been shown to hold promise in detecting early cancers (Hoque *et al*, 2006).

There are several advantages for the detection of aberrant DNA methylation over the genetic methods discussed above. First, hypermethylation of multiple tumour suppressor genes are frequently observed in cancers. Thus, the sensitivity of a cancer detection test can be enhanced by simultaneous detection for the hypermethylation of multiple genes. Second, using methylation-specific PCR, the detection of aberrantly methylated sequences in plasma can be achieved even when these sequences only constitute a minor portion of the circulating DNA. Thus, it is expected to be more sensitive than the detection of microsatellite alterations, which can only be detected when the circulating DNA is mainly comprised of tumoral DNA. Moreover, in contrast to the detection of viral DNA that is only useful for virus-associated cancers, hypermethylation of tumour suppressor genes is widely observed in different types of cancers and, hence, the detection of these changes in plasma can be applied to a wide range of cancers.

### Tumour-associated RNA transcripts

Cancer cells often have distinct gene expression patterns from normal tissues. This difference can be used diagnostically through detecting the tumour-specific transcripts in the circulation of cancer patients. Early studies mainly utilised these tumour-specific mRNA as a marker for circulating tumour cells and, hence, the detection of such transcripts in peripheral blood cells was mostly reported in patients with distant metastases. In 1999, the detection of tyrosinase RNA was reported in the cell-free plasma/serum of patients suffering from melanoma (Kopreski *et al*, 1999). These findings were surprising because mRNA molecules are generally thought to be extremely labile in plasma/serum. A previous study demonstrated that exogenous mRNA spiked into plasma would be rapidly degraded within seconds (Tsui *et al*, 2002). The stability of circulating endogenous RNA molecules was later shown to be because of their association with small particles in plasma, which might protect them from the degradation by circulating ribonucleases (Ng *et al*, 2002).

The demonstration of tumour-associated RNA transcripts in cell-free plasma/serum not only provides new targets for cancer detection but also opens up the possibility of non-invasive gene expression profiling for cancers. Recently, Li *et al* (2006) performed gene expression profiling using serum RNA from patients suffering from oral cancers and compared them with the expression profiles of healthy subjects. They identified over 300 transcripts that were differentially expressed in the serum of cancer patients and controls and they showed that the difference in the expression patterns can accurately distinguish the two groups (Li *et al*, 2006). Although this initial investigation on non-invasive gene expression profiling using serum RNA is very exciting, these results have to be interpreted with caution. In a previous study, it was shown that majority of the circulating DNA was derived from haematopoietic cells (Lui *et al*, 2002). Although there is no existing information on the origin of circulating RNA, it is logical to believe that a significant amount of circulating RNA also comes from the haematopoietic system because of their close proximity. In this regard, it is unclear whether the difference in gene expression profiles between cancer patients and healthy control subjects is the response of blood cells to a disease condition or is a direct reflection of gene expression in the cancer cells. If it is the former situation, the choice of control group would be very important as this would determine whether the differences in transcription patterns are specific for the type of cancer or they are just non-specific changes to disease in general, for example inflammatory responses.

### Alterations in physical properties

In addition to the genetic and epigenetic changes, changes in the physical properties of circulating nucleic acids have been observed in cancer patients. In 1989, Stroun *et al* demonstrated reduced strand stability of the circulating DNA in cancer patients (Stroun *et al*, 1989). This feature was also observed in DNA extracted from malignant tissues. Their findings represent the earliest evidence for the presence of circulating tumour-derived DNA in cancer patients. Recently, the size of circulating nucleic acids has also been found to be altered in patients suffering from malignant disease. Interestingly, the changes in integrity for RNA and DNA are opposite. Thus, Umetani *et al* (2006) reported that serum DNA integrity was significantly higher in patients with stage II, III and IV breast cancer than in healthy females. The increase in serum DNA integrity was also shown to predict tumour progression (Umetani *et al*, 2006). On the other hand, Wong *et al* (2006) reported that the integrity of circulating RNA was significantly reduced in NPC patients (Wong *et al*, 2006), and the RNA integrity was lower in patients with stage III and IV disease when compared with stage I and II patients. Interestingly, these aberrations in RNA integrity could be normalized after treatment (Wong *et al*, 2006).

The precise mechanisms leading to the alterations in circulating nucleic acids integrity remain unclear. It has been postulated that, in healthy subjects, circulating DNA is mainly derived from the apoptotic cells while, in cancer patients, DNA is released from dead cancer cells that have not gone through the normal apoptotic process (Umetani *et al*, 2006). This postulation would explain why the circulating DNA in healthy subjects is similar to the size of nucleosomal DNA, i.e. 180 bp, (Chan *et al*, 2004) and the size of circulating DNA in cancer patients is longer (Umetani *et al*, 2006). On the other hand, circulating RNA molecules are more susceptible to degradation than DNA molecules and the ribonuclease activity has been reported to be increased in the plasma of cancer patients (Reddi and Holland, 1976). Therefore, the reduced integrity of plasma RNA in cancer patients has been postulated to be a result of accelerated RNA degradation by circulating ribonucleases (Wong *et al*, 2006). Interesting, the alterations of the integrity of circulating nucleic acids could also be observed in other non-malignant conditions, for example pregnancy (Chan *et al*, 2004).

## CONCLUSIONS

The study of circulating nucleic acids for clinical diagnostic purposes is a new but rapidly expanding field. The preliminary reports on the detection of cancer-associated changes in the serum/plasma of cancer patients are promising. However, there are challenges ahead for adapting these tests for routine clinical use. First, as most of the existing reports aimed to explore new potential markers and involved relatively few patients, the results of different studies using similar technologies and gene targets could be quite different. Therefore, large-scale studies would be necessary to address the sensitivities, specificities and the clinical usefulness of individual markers before these tests could be used for the management of patients. Second, there is currently no consensus on the sample type and sample preparation protocols for circulating nucleic acid analysis. Preanalytical factors, including the use of anticoagulants, storage duration and conditions, have been shown to affect the quantity and quality of circulating nucleic acids (Chan *et al*, 2005b). Therefore, standardisation of these preanalytical factors would be an important issue if large-scale international studies are to be carried out. Third, efforts are required to minimise the chance of contamination and, thus, false-positive results. PCR is the most common technique used in molecular diagnosis because of its ability in amplifying DNA and detecting a DNA target at a low concentration. However, its high sensitivity also poses potential risks for carry-over contamination. The most important strategy to minimise this problem is to physically separate the areas used for preparing the PCRs and handling PCR products and this should be taken into consideration at an early stage of establishing a molecular diagnostic laboratory. Moreover, the use of technologies that do not require post-PCR handling, for example real-time PCR, is also useful for reducing the chance of contamination. Overall, we are optimistic that circulating nucleic acid analysis would become an important tool for the clinical management of cancer patients in the near future.
